# Noninsect Arthropods in Popular Music

**DOI:** 10.3390/insects2020253

**Published:** 2011-05-26

**Authors:** Joseph R. Coelho

**Affiliations:** Biology Program, Quincy University, 1800 College Ave., Quincy, IL 62301, USA; E-Mail: coelhjo@quincy.edu; Tel.: +1-217-228-5432 ext. 3268; Fax: +1-217-228-5222.

**Keywords:** arthropod, crustacean, crab, shrimp, lobster, spider, scorpion, music, cover art

## Abstract

The occurrence of noninsect arthropods in popular music was examined in order to explore human attitudes toward these species, especially as compared to insects. Crustaceans were the most commonly referenced taxonomic group in artist names, album titles and cover art, followed by spiders and scorpions. The surprising prevalence of crustaceans may be related to the palatability of many of the species. Spiders and scorpions were primarily used for shock value, as well as totemic qualities of strength and ferocity. Spiders were the most abundant group among song titles, perhaps because of their familiarity to the general public. Three noninsect arthropod album titles were found from the early 1970s, then none appear until 1990. Older albums are difficult to find unless they are quite popular, and the resurgence of albums coincides with the rise of the internet. After 1990, issuance of such albums increased approximately linearly. Giant and chimeric album covers were the most common of themes, indicating the use of these animals to inspire fear and surprise. The lyrics of select songs are presented to illustrate the diversity of sentiments present, from camp spookiness to edibility.

## 1. Introduction

Cultural entomology has examined the relationship between humans and insects through a variety of popular media. For example, with notable exceptions, insects in film are used to inspire fear [1–3]. Recent efforts focused on the place of insects in popular music demonstrated that they are frequently used for aesthetic value, shock value, and perceptions of strength and power [4–6].

In the present study, I examine taxonomy and themes in popular music to determine the manner in which people perceive noninsect arthropods. This group was chosen as a sister group to the insects as a means of testing whether similar trends occur. It was anticipated that arthropods with well established reputations, such as scorpions and black widows (*Latrodectus* spp.), would be used as means to project ferocity and toughness. Spiders are rated the ugliest among a variety of insects, and are second only to rats in fear-inducement [7]. In cartoons (comic strips), spiders are by far the most abundant among noninsect arthropods [8].

## 2. Experimental Section

Amazon.com^®^ was searched for popular terms referring to noninsect arthropods (Table 1). Recorded were the names of artists and albums which matched the terms and appeared to be intended as biological. The original year of issue of the album, if available, was also recorded. Matching albums and those of matching artists were examined further to discover whether cover art depicting noninsect arthropods was present.

Limits established for previous studies [5,6] were largely followed for artist names and album titles. Note that a group of songs, issued as a single body of work, is referred to as an album irrespective of the medium prevalent at the time of its issuance (vinyl LP, CD, *etc.*). As matching song titles were too numerous to justify close examination (e.g., >4000 for “spider”), only the number of hits on each search term was recorded (on March 26, 2010). The searches were conducted from October 2008 to March of 2010. All statistics were performed using Microsoft^®^ Excel v. 9.0. Each album was classified by taxonomic group according to a recent scheme [9]. If more than one group was shown, all were counted. Specific attributes of the animals shown were noted, including whether they appeared to be very large (giant), in great numbers (numerous), were depicted as tattoos, were presented as food, or shared arthropod and human features (chimeric).

For much of the taxonomic analysis, data were lumped according to three convenient groups: Crustacea, Araneae and Scorpiones. Although not taxonomically equivalent, these groups are readily recognizable to the general public. Other groups, with many fewer results, were not considered in great detail.

## 3. Results and Discussion

### 3.1. Taxonomy

Noninsect arthropods were quite rare in popular music. Insects are represented by 160% more artists and 59% more albums than noninsect arthropods. Insect-specific music was estimated to comprise only 0.1% of the pop music canon (see [5]), suggesting that there is but miniscule interest in the other arthropods. Nonetheless, sample sizes were sufficient to examine human attitudes toward those noninsect arthropods that do appear in popular music.

**Figure 1:**
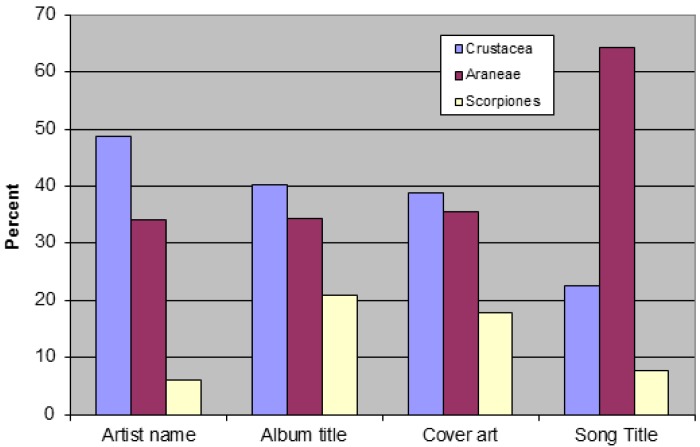
Simplified taxonomic summary of noninsect arthropods in popular music.

There were 82 artist names related to noninsect arthropods, while there were 134 such album titles, 62 cases of cover art and 7352 songs. A list of artists and albums is provided in the supplementary material. Crustaceans were the most commonly encountered group among artist names, album titles and album covers, followed by spiders then scorpions (Figure 1). In fact, crustaceans were represented in almost 50% of artist names and approximately 40% of album titles and covers. However, among song titles, spiders were by far the most abundant group (66%), followed by crustaceans (23%) and scorpions (8%). Crustaceans were surprisingly abundant relative to other noninsect arthropods in popular music. The prevalence of crustaceans was unanticipated (crustaceans were not even noted in comic strips [8]. Edible crustaceans comprised the majority of those mentioned, including crab, lobster, shrimp and crayfish. Barnacles (generally considered inedible) were the only other crustacean to garner significant numbers. These trends may reflect a preoccupation with food. This theme was seldom encountered among studies of insects and music, perhaps because insects are rarely consumed in the USA.

Indeed, the food theme was reflected rather directly in crustacean artist names, such as Jumbo Shrimp Band, Plate O' Shrimp, and Lobster Newberg. Albums of edible crustaceans are found in *King Crab, Crabs in a Bucket, Crab Rangoon, Catfish Shrimp-N-French Fries, All New With Shrimp Cocktail, She Invited Me for Lobster,* and *How Do You Like Your Lobster?* The only overtly negative crustacean examples were the artist Hell Crab City and the albums *A Giant Crab Comes Forth* and *Cannibal Crabs Crawl to Kill*. Visual representations of arthropods as food were relatively rare in cover art. Crustaceans were a distant second to spiders in song titles as well. These trends suggest that the arthropods we choose to depict in words or songs differ from those we choose to depict in art. A similar effect was observed among insects, where Hymenoptera dominate artist names, album titles, and songs, but Lepidoptera are most common on cover art. In the case of insects the shift is obviously because of the greater visual appeal of butterflies and moths. In noninsect arthropods, the reasons are less clear. Spiders dominate song titles, perhaps because of their everyday familiarity and fear-inducing potential. Food may be fun to sing about, but it may not make an album cover that sells well.

The crustacean connection runs deep in rock history, as the Crawdaddy Club was a well known venue in England where, incidentally, the Rolling Stones performed very early in their career. The club's name inspired that of *Crawdaddy!*, which is claimed to be the first magazine of rock music criticism, and still exists in online form [10].

Primarily negative attitudes toward spiders were depicted by the artist names, such as Mean Red Spiders, Spider Virus, and Spider Vomit. There were also four variations of black widow and two of brown recluse in artist names. Albums *Spin*, *Spider*, *Spin* and *Along Came a Spider* project a certain creepiness, while 10 black widow albums and three of brown recluse provide further evidence of the unsurprising adoption of spiders for shock value. *Arachnophobiac*, however, is balanced by *Arachnomania*.

The greatest single contributor to the data was German classic rock group The Scorpions, with 16 album covers and three titles of their own, as well as two by a former member and four tribute albums (each with scorpion cover art) by other artists.

Although data were lumped into groups for analysis of major trends, it is interesting to note that some relatively obscure species were represented in song titles (Table 1), including vinegaroon (by alternative group Calexico) and myriapod (by progressive rock group Ozric Tentacles).

### 3.2. Chronology

**Figure 2:**
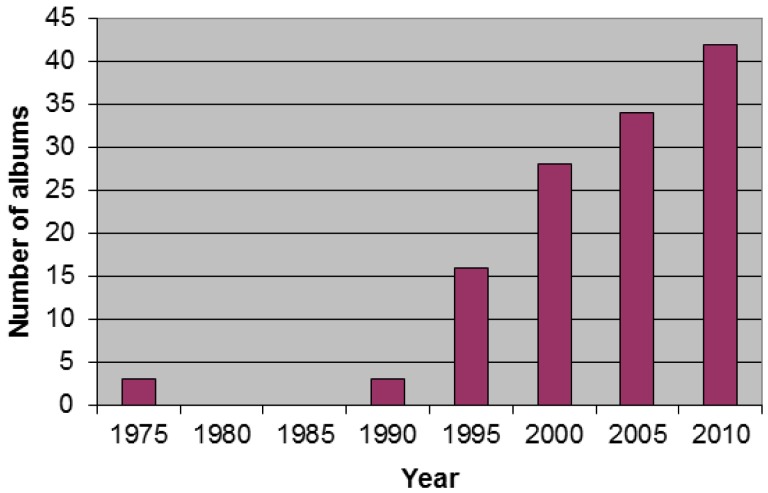
Number of noninsect arthropod albums released over time.

The first noninsect arthropod albums appear in the early 1970s. No further examples appear until 1990, when such works increase in frequency approximately linearly to the present (Figure 2). The increasing number of arthropodic albums would seem to indicate a concomitant interest in these species. This temporal trend is similar to that observed among insects in music [5] and cockroaches in film [2] and suffers from some of the same biases. More recent albums are overrepresented in online sales, as older material does not sell as well. The total number of album releases is likely to be increasing at a rate that, being unknown, cannot be controlled for. The earliest noninsect arthropod album I could find was Noggins's *Crab Tunes* from 1971. David Bowie's *The Rise and Fall of Ziggy Stardust and the Spiders from Mars* followed in 1972, then Jimmy Buffet's classic *A White Sport Coat and a Pink Crustacean* in 1973. These albums illustrate the difficulty of finding older releases. Noggins's *Crab Tunes* was mentioned by in a volume on music history [11], but is apparently out of print. Bowie's *Spiders* is quite well known and presumably still sells. Jimmy Buffett's *Crustacean* made it into the data mostly because of the author's prior awareness of it, though it is still available. The early history of musical insect groups was recently examined [12], and only one noninsect arthropod group was mentioned. Known as Webb and His Spiders, it is not clear that they ever released any albums, and they dated from the 1930s, well before the rock-and-roll era [13]. It is curious that no albums related to noninsect arthropods issued between 1973 and 1990 could be found, though the resurgence of releases is strikingly coincident with the timing of the rise of the internet. It is probable that such albums exist, but are not well documented in online sources. Similar gaps are noted in the issuance of arthropodic films [1].

### 3.3. Cover Art

**Figure 3:**
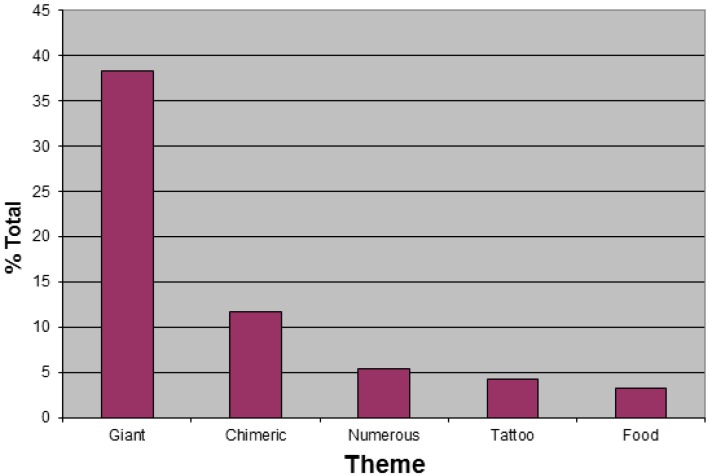
Number of noninsect arthropod album covers with selected themes depicted.

Gigantism was the most common theme in cover art, followed by chimeric figures, infestations, tattoos and food (Figure 3).

It is not surprising that Gigantism was a frequent theme in cover art, as noninsect arthropods are frequently used for shock value. Gigantism enhances the scariness of such depictions, just as it does in film [1]. Many of the giants were scorpions, primarily by the artist of the same name. A notable exception was a colorful, photorealistic centipede which fills the front of the Bishop album *Centipede*. The anterior end is featured on the back. The most directly threatening image comes from *Cannibal Crabs Crawl to Kill* (by Various Artists), and requires no exaggeration in size of the largest terrestrial arthropods in the world [14]. It apparently depicts a pair of coconut crabs (*Birgus latro* L.) attacking a man, who is attempting to fend them off with a club. Although they are omnivorous [9,15], coconut crabs are not known to attack humans.

**Figure 4:**
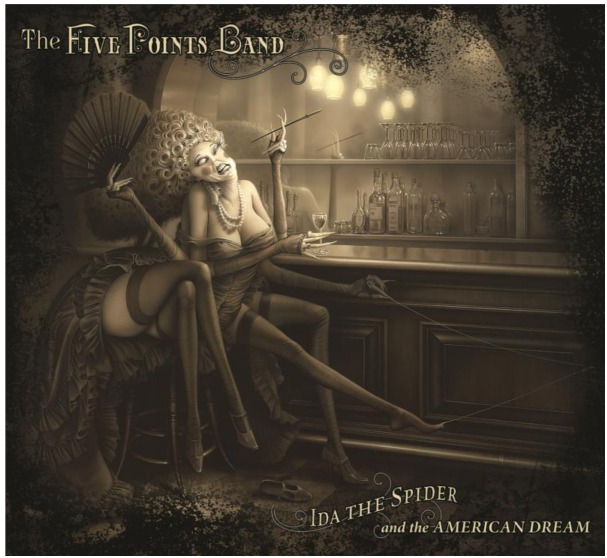
Cover art from The Five Points Band's *Ida the Spider and the American Dream*. Copyright Kirk Reinert; used by permission.

A similar negative effect applies to depictions of numerous individuals, as infestations are more impressive than individual animals. However, chimeric arthropods were the second most common theme. Chimeric, or at least anthropomorphic, insects are common in animated films that feature insects [3]. Although chimeric insects are common on album covers, the explanation was generally the fantasy of human flight, as the most common chimeric organism was a human with lepidopteran wings [6]. This explanation fails in the case of noninsect arthropods, which cannot fly. In this case, shock value may again explain the theme. Human-arthropod chimeras are regarded as particularly horrible (e.g., various versions and sequels of the film *The Fly*). Few of those found on album covers could be said to be appealing. A typical example is The Scorpions *Savage Amusement*, showing a woman with a scorpion's tail. In some cases, the chimera was used to promote a metaphor. A striking example is The Five Points Band's *Ida the Spider and the American Dream*, wherein an apparent human-black widow chimera sits at a bar with her four human arms and four human legs (Figure 4). One hand and foot are attached to webs, while her hair resembles an egg mass. The implication may be that she is waiting for her next victim, as it is well known that the black widow female may eat the male [16]. Only four tattoos of noninsect arthropods were shown on album covers, though insect tattoos may be relatively frequent, and rock artists are frequently heavily tattooed. Spiders and scorpions are the most common among noninsect arthropods in tattoos [17]. However, their representation in cover art is low among arthropods, insect and otherwise.

### 3.4. Lyrics

Although most noninsect arthropod songs are relatively obscure, a few have been sufficiently successful to enter the popular conscience. Examination of lyrical content may reveal further insight into the noninsect arthropod perception by humans (performances of nearly all songs below are available for viewing on YouTube^®^). Recently, No Doubt's 1995 single “Spiderwebs”, from *Tragic Kingdom*, celebrates the arachnid's handiwork, but only in the metaphorical sense, as revealed in the relevant passage:

And now I'm stuck in the web

You're spinning

You've got me for your prey

Sorry, I' m not home right now

I'm walking into spiderwebs

Another very popular tune was the B-52's “Rock Lobster” from their 1978 self-titled debut. Though the lyrics defy ready interpretation, there is no question that the song is fun and extremely danceable. The crustacean is mentioned in the first two stanzas:

We were at a party

His ear lobe fell in the deep

Someone reached in and grabbed it

It was a rock lobster

We were at the beach

Everybody had matching towels

Somebody went under a dock

And there they saw a rock

It wasn't a rock

It was a rock lobster

Alice Cooper's “The Black Widow”, from the classic *Welcome to My Nightmare* (1975), begins with a monologue voiced by horror film star Vincent Price:

Leaving Lepidoptera…Please, don't touch the display, little boy, aha cute! Moving to the next aisle we have Arachnida, the spiders, our…finest collection. This friendly little devil is the heptothilidi, unfortunately harmless. Next to him, the nasty *Lycosa raptoria*, his tiny fangs cause creeping ulcerations of the skin (laugh). And here, my prize, the Black Widow. Isn't she lovely? …And so deadly. Her kiss is fifteen times as poisonous as that of the rattlesnake. You see her venom is highly neurotoxic, which is to say that it attacks the central nervous system causing intense pain, profuse sweating, difficulty in breathing, loss of consciousness, violent convulsions and, finally…death. You know what I think I love the most about her is her inborn need to dominate, possess. In fact, immediately after the consummation of her marriage to the smaller and weaker male of the species she kills and eats him…(laugh) oh, she is delicious…And I hope he was! Such power and dignity…unhampered by sentiment. If I may put forward a slice of personal philosophy, I feel that man has ruled this world as a stumbling demented child-king long enough! And as his empire crumbles, my precious Black Widow shall rise as his most fitting successor!

This narration, of debatable accuracy, is followed by the actual song (first two stanzas only shown):

These words he speaks are true

We're all humanary stew if

We don't pledge allegiance to

The Black Widow

The horror that he brings

The horror of his sting

The unholiest of kings

The Black Widow

Among the glaring errors are the sting instead of a bite, and the character being portrayed as a male, but perhaps Cooper is using the Black Widow as a metaphor for a despotic ruler or even an addictive drug.

Although most fans do not take Alice Cooper too seriously, The Who's “Boris the Spider” definitely falls into the scary/campy category:

Look, he's crawling up my wall

Black and hairy, very small

Now he's up above my head

Hanging by a little thread

Boris the spider

Boris the spider

Now he's dropped on to the floor

Heading for the bedroom door

Maybe he's as scared as me

Where's he gone now, I can't see

Boris the spider

Boris the spider

Creepy, crawly

Creepy, crawly

Creepy, creepy, crawly, crawly

Creepy, creepy, crawly, crawly

Creepy, creepy, crawly, crawly

Creepy, creepy, crawly, crawly

There he is wrapped in a ball

Doesn't seem to move at all

Perhaps he's dead, I'll just make sure

Pick this book up off the floor

Boris the spider

Boris the spider

(repeat chorus)

He's come to a sticky end

Don' t think he will ever mend

Never more will he crawl ‘round

He's embedded in the ground

Boris the spider

Boris the spider

As with insects [5], descriptions of noninsect arthropod control methods, no matter how crude, are rare in pop music. Nonetheless, the end of Boris reflects how many people feel about spiders in general. Uncharacteristic of The Who's work, the song is well known to Who fans, a rare contribution by bass player John Entwhistle and appears on *A Quick One* (1966).

Perhaps lesser known songs hold more insights than the popular ones. The palatability of crustaceans is reflected clearly in Elvis Presley's “Crawfish”, a 2-minute duet (with Kitty White) from the movie *King Creole* (1958). Though the original was performed in a traditional style, a cover version by the Bonedaddys in 1989 was rewritten to last longer (5:34 min) and rock harder. The lyric even has instructions for the capture and preparation of this common, freshwater crustacean.

Well I went to the bayou just last night

There was no moon but the stars were bright

Put a big long hook on a big long pole

And I pulled Mr. Crawfish out of his hole

Crawfish

See I got him, see the size

Stripped and cleaned before your eyes

Sweet meat look, fresh and ready to cook

Crawfish

Now take Mr. Crawfish in your hand

He's gonna look good in your frying pan

If you fry him crisp or you boil him right

He'll be sweeter than sugar with every bite

Crawfish

## 4. Conclusions

Human attitudes toward noninsect arthropods are demarcated on largely taxonomic lines. Crustaceans, most abundant among artist names, album titles, and cover art, are frequently presented in a favorable light as food items. Spiders, by far the most common among song titles, are used for shock value and totemic attributions of power. Scorpions, intermediate in popularity in all categories, are almost exclusively used to induce fear. Noninsect arthropodic music may be increasing in frequency, but sampling biases abound. In contrast to names and titles, themes prevalent among album covers featuring noninsect arthropods tend toward the frightening and horrific, demonstrating a difference between written and visual forms.

## Figures and Tables

**Table 1:** Number of musical references to noninsect arthropod keywords.

	**Artist**	**Album**	**Cover**	**Song title 1**
**Subphylum Chelicerata** **Class Arachnida** **Order Scorpiones**				
Scorpion	5	28	11	566
**Order Uropygi**				
Vinegaroon	0	0	0	5
**Order Araneae**				
Arachnid	0	0	0	32
Spider	19	28	13	4041
Black Widow	6	10	5	398
Arana	0	2	2	236
Brown recluse	2	3	0	12
Funnel web	0	1	1	2
Orb Weaver	0	0	0	3
**Order Opiliones**				
Daddy Long Legs	1	4	1	64
**Order Acari**				
Mite	4	0	1	133
**Subphylum Mandibulata** **Class Myriapoda**				
Myriapod	0	0	0	13
Millipede	1	1	1	10
Centipede	3	1	2	173
**Class Crustacea**				
Barnacle	7	4	0	113
Crustacean	2	6	1	22
Crab	10	12	9	773
Crayfish^2^	7	2	1	23
Isopod	0	0	0	0
Krill	1	0	0	22
Lobster	8	17	9	354
Scud	0	1	0	62
Shrimp	5	10	4	295

1 Number of hits for Amazon.com^®^ search on song title.

2 Combined totals for crayfish, crawfish, crawdad and crawdaddy.

## References

[1] Mertins J.W. (1986). Arthropods on the screen. Bull. Entomol. Soc. Am..

[2] Berenbaum M.R. (1996). Roach clips and other short subjects. Am. Entomol..

[3] Leskosky R.J., Berenbaum M.R. (1988). Insects in animated films. Not all “Bugs” are bunnies. Bull. Entomol. Soc. Am..

[4] Berenbaum M.R. (1996). “Let me tell you ‘bout the birds and the bees….”. Am. Entomol..

[5] Coelho J.R. (2000). Insects in rock and roll music. Am. Entomol..

[6] Coelho J.R. (2004). Insects in rock and roll cover art. Am. Entomol..

[7] Hardy T.N. (1988). Entomophobia: The case for Miss Muffet. Bull. Entomol. Soc..

[8] DeJong G.D. (1994). Insect cartoons: When do they appear in newspapers and magazines?. Am. Entomol..

[9] Pechenik J.A. (2010). Biology of the invertebrates.

[10] Crawdaddy! http://www.crawdaddy.com/.

[11] Pareles J., Romanowski P. (1983). The Rolling Stone Encyclopedia of Rock & Roll.

[12] Clarke S. (2010). Catch that buzz: A brief history of musical insect groups, part 1. Am.Entomol..

[13] Sanders J. (2005). PROFILE: Preston H. Love. http://www.nsea.org/news/LoveProfile.htm.

[14] Brown I.W., Fielder D.R. (1991). The Coconut Crab: Aspects of the Biology and Ecology of Birgus latro in the Republic of Vanuatu.

[15] Wilde J.E., Linton S.M., Greenaway P. (2004). Dietary assimilation and the digestive strategy of the omnivorous anomuran land crab *Birgus latro* (Coenobitidae). J. Comp. Physiol. B.

[16] Jones S.C. Black widow spider fact sheet. http://ohioline.osu.edu/hyg-fact/2000/2061A.html.

[17] Pearson G.A. (1996). Insect tattoos on humans: A “dermagraphic” study. Am. Entomol..

